# Evaluation of bio-efficacy and durability of long-lasting insecticidal nets distributed by malaria elimination programme in Eastern India

**DOI:** 10.1186/s12936-020-03260-2

**Published:** 2020-05-24

**Authors:** Sudhansu Sekhar Sahu, Amol Vasantrao Keshaowar, Sonia Thankachy, Dilip Kumar Panigrahi, Premalata Acharya, Vijayakumar Balakrishnan, Ashwani Kumar

**Affiliations:** Indian Council of Medical Research-Vector Control Research Centre, Medical Complex, Indira Nagar, Puducherry, 605006 India

**Keywords:** Attrition, Bio-efficacy, Long-lasting insecticidal nets, Physical integrity, Survivorship

## Abstract

**Background:**

Long-lasting insecticidal nets (LLINs) are the most favoured vector control tools worldwide. Timely monitoring and evaluation of LLINs is important to sustain the impact of this promising vector control method and for replacement of worn-out and those rendered ineffective. During the mid-2017, LLINs were distributed by the National Vector Borne Disease Control Programme (NVBDCP) in high malaria endemic districts of the eastern coastal state of Odisha. The study was carried out to assess the field performance of the LLINs post 30 months of distribution in Koraput district of Odisha state.

**Methods:**

A total of 130 households were randomly selected from three villages of Laxmipur CHC in Koraput district, Odisha, India; one each from hilltop, foothill and plain terrain. The net users were interviewed to elicit information on usage, washing practices, physical integrity, bio-efficacy and survivorship of LLINs to confirm the claimed three-year life of the LLINs.

**Results:**

74.8% of the LLINs were physically present after 30 months of distribution. The numbers (%) of LLINs used previous night varied from 30 to 61% between study villages. 74% respondents were using the LLINs throughout the year and 26% only seasonally. Of the total, 85% of the nets were reported to be washed and 95% nets were dried under shade as recommended. Altogether, 58% of the surveyed nets were found torn with holes. Of these, 74 (57%) nets were in good condition, 10 (8%) nets were in serviceable and 45 (35%) nets were badly torn and needed replacement. A total of 45 (93.75%), 68 (80%) and 71 (63.8%) LLINs were physically present in hilltop, foothill and plain villages, respectively. The LLINs did meet the efficacy criteria, given the 100% mortality to the exposed *Anopheles jeyporiensis* mosquitoes post 30 months distribution.

**Conclusions:**

The findings of this study were communicated to the programme officials of the state and LLINs were replenished soon after 31st month post-distribution of LLINs.

## Background

Mass distribution of long-lasting insecticidal nets (LLINs) in India follows a worldwide established successful strategy for control of malaria [1, 2]. Unlike conventional insecticide-treated nets (ITNs), which loses the effectual insecticidal content after 1–2 washes and requires re-treatment after every 6–12 months, LLINs are designed to sustain the physical barrier for 3 years in household conditions and can retain the residual efficacy up to 20 washes [3]. Physical integrity and survivorship are two important indicators determining the regularity of the mass distribution campaigns of LLINs. The poor physical condition of the net may offer little-to-no protection to the users and then survivorship data alone will, most likely, underestimate net loss. Questions have arisen about whether LLINs retain the durability (survivorship, fabric integrity and bio-efficacy) up to 3 years in different eco-epidemiological settings, where LLINs are distributed with universal coverage by the malaria control programme in different countries [4]. Therefore, the World Health Organization (WHO) recommends countries to monitor by quantifying three indicators: survivorship, fabric integrity and bio-efficacy to assess durability of LLINs following the mass distribution campaigns [5, 6].

In India, the National Vector Borne Disease Control Programme (NVBDCP) has gradually scaled up LLIN distribution to control malaria in the endemic states since 2009 [2]. To maintain the impact of this vector control strategy, timely monitoring and evaluation of LLINs is considered most important which helped the malaria control programme for timely replacement of worn out nets [7, 8]. Global studies conducted elsewhere revealed that the physical integrity and survivorship of LLINs show discordance from 2 years or above [9–11]. The dissimilarity in findings may be due to the sociological, behavioural, environmental factors and the brand of the LLIN [6, 12, 13]. Therefore, updating information on the bio-efficacy and fabric integrity of field distributed LLINs and usage practices by the community at different time intervals is critical for choice of an appropriate LN for the mass distribution programme.

Odisha state contributed 40.9% of the total malaria incidences and 23.3% of the total malaria mortalities in India during 2016 and 41.2% of total malaria incidences and 12.4% of total deaths due to malaria during 2017 [14]. From 1958 onwards, the main vector control intervention adopted in the state was indoor residual spraying (IRS) with DDT, two rounds per year [15, 16]. Since, the strategy could not make any tangible impact on malaria incidences during the past few years, mass distribution of 11.3 million LLINs was done in 17 high endemic districts of the state in mid 2017 under National Strategic Plan for malaria elimination [17, 18]. Thereafter, a significant reduction in malaria cases was observed in the state [18]. However, the reduction did not show a sharp declining trend. This may be due to the impact of LLINs on malaria morbidity and mortality, which depends mainly on the usage of mosquito nets and their durability (survivorship, fabric integrity and bio-efficacy). So far, there is little information available regarding the durability, washing practices and usage rate of LLINs distributed en-masse by the malaria control programme in India. In this study, the durability of LLINs distributed by NVBDCP was assessed among the tribal inhabitants of Odisha state after 30 months of field use.

## Methods

### Study area

The study was undertaken in Laxmipur Community Health Centre (CHC) of Koraput district, Odisha state. The CHC has a population of about 66,935, (as per 2011 census) of which 67% are tribes living in 176 villages and hamlets under 16 sub-centres. Most of the villages in this CHC area are inaccessible (situated on hilltops, foothills) and are connected by narrow jungle or hilly tracks, which are accessible only on foot [2]. From the year 1958 onwards, IRS with DDT was the sole vector control intervention ongoing in the Laxmipur CHC. From 2009, distribution of LLINs was done in many high endemic areas of the CHC in a phase wise approach [19]. As a part of malaria elimination programme in India, during mid-2017, mass distribution of PermaNet 2.0 LN, a 100-denier (40 g/m^2^ fabric weight) polyester net (size: 180 × 120 × 150 cm) coated with deltamethrin (55 mg/m^2^) was carried out in 17 high endemic districts of Odisha state including Koraput district. Under this programme, LLINs were distributed free of cost in these villages during July–August 2017. The annual parasite incidence (API) of Laxmipur CHC was 55.3 in 2015, 58.2 in 2016, 63.6 in 2017, 6.4 in 2018 and 4.1 in 2019, respectively. (*Source*: CDMOs Office, Koraput) (Table 1). *Anopheles fluviatilis* and *Anopheles culicifacies* are the two main vectors of malaria in the study area [2, 16]. *Anopheles fluviatilis* prefers to rest in human dwellings and is highly anthropophagic. *Anopheles culicifacies* rest in cattle sheds and prefers to feed on cattle. Streams and terraced paddy fields are the major breeding habitats of *A. fluviatilis*, whereas, *A. culicifacies* prefers to breed in riverbed pools, terraced paddy fields and ponds [2, 16]. The infection rate of *A. fluviatilis* and *A. culicifacies* in Koraput district was 7.8 and 1.07, respectively [16].

**Table 1:** Epidemiological data of malaria in Laxmipur CHC before and after LLIN distribution

Year	Population	BSC	Positives	*Pf*	*Pv*	Deaths	SPR	*Pf* %	API
2015	67607	25518	3737	3605	132	1	14.6	96.4	55.3
2016	68641	29687	3994	3819	175	0	13.4	95.6	58.2
2017	69902	29091	4449	4238	211	0	15.29	95.2	63.6
2018	71300	23292	457	428	29	0	1.96	93.6	6.4
2019	72726	25815	299	293	6	0	1.15	97.9	4.1

*Pf, Plasmodium falciparum, Pv, Plasmodium vivax,* SPR, slide positivity rate

API, annual parasite incidence

From the CHC, three villages (Ramijhola (19^°^.96′21′′ N latitude and 83^°^.22′88′′ E longitude), Keskapadi (19^°^.12′89′′ N latitude and 82^°^.22′36′′ E longitude) and Bandikar (19^°^.00′75′′ N latitude and 83^°^.14′73′′ E longitude) were randomly selected representing the three ecotypes, hilltop (TH), foothill (FH) and plain (PL), respectively. Total population of these villages was 2461 residing in 533 holdings.

### Sample size and study design

Sampling of households was done based on the total number of households in the selected village. The sample size was estimated by assuming 40% usage rate and an error margin of 10% with 95% confidence interval (CI). Thus, a total of 130 holdings covering 538 populations were selected for conducting the surveys. Based on the proportion of households available in each village, a total of 130 households were selected; 27, 49 and 54 households from TH, FH and PL villages of Laxmipur CHC, respectively. A random sampling survey was undertaken during February and March 2020. The household heads were interviewed by door-to-door visits. One used LLIN in each selected 130 holdings was inspected and the information was recorded.

A structured questionnaire prepared by the research team of Indian Council of Medical Research-Vector Control Research Centre (VCRC) Puducherry was used for the survey which consists of the following parts: general information; status of distributed LLINs, physical presence/absence of nets, usage rate, physical integrity, net utilization pattern/frequency of use and washing practices.

### Assessment of fabric integrity

One net from each of the selected households (n = 129) were examined physically for the presence of holes and if present, the number of holes were counted and size and location of the holes (top, upper side, lower side), open seams, burns and tears for each net was recorded. The holes were grouped into four categories as per the size, viz. size 1: 0.5 cm–2 cm; size 2: 2 cm–10 cm; size 3: 10 cm–25 cm; size 4: > 25 cm and having weight of 1, 23, 196 and 576, respectively [3]. The hole index was calculated as: (1 × number of holes of size 1) + (23 × number of holes of size 2) + (196 × number of holes of size 3) + (576 × number of holes of size 4) [3]. The hole area was calculated as 1.23, 28.28, 240.56 and 706.95, respectively for each hole of size 1, 2, 3 and 4 [3]. For each net, the hole area was calculated as: (1.23 × number of holes of size 1) + (28.28 × number of holes of size 3) + (240.56 × number of holes of size 3) + (706.95 × number of holes of size 4) [3]. Based on the proportionate hole index (pHI) obtained, LLINs were categorised to one of the three conditions: pHI ≤ 64—good; pHI ≤ 642—serviceable; pHI > 642—replace [20].

### Net survivorship and attrition

The physical presence/absence of the LLINs in the selected 130 households after 30 months of distribution was recorded. Information on the disposal of LLINs due to usual wear and tear, selling, gifting or stealing the nets and usage of LLINs for other purposes was collected. Attrition rate (percentage of LLINs lost due to discarding of nets because of excessive loss of fabric integrity, movement of nets by selling them, migration or misuse) was calculated as

$$\frac{\text{Total LLINs lost in the selected households}}{\text{Total number of LLINs distributed to surveyed households}} \times 100.$$

### Informed consent and ethical clearance

For conducting the net survey, written consent was obtained from the selected household heads. Prior ethical clearance was obtained from the Human Ethics Committee of the ICMR-VCRC Puducherry for interviewing the household heads/net users.

### Bio-efficacy of LLINs

Insecticidal efficacy of LLINs was evaluated using cone bio-assays following WHO guidelines 2013, at the beginning (September 2017) and at every 6 month intervals up to 30 months (February 2020) of net distribution. Each time, a total of six LLINs with different washing frequencies were randomly selected and withdrawn from the different villages of Laxmipur CHC for conducting the cone bio-assays. These nets were replaced with new LLINs and the holdings were not included for bio-assays in future. The net pieces (30 × 30 cm) were cut from 5 locations from each of the six withdrawn net as per WHOPES sampling scheme [3]. The data on washing frequencies of sampled LLINs were also collected. Since, adequate number of susceptible *An. fluviatilis* mosquitoes was not available in the field; bio-assays were carried out with wild caught pyrethroid susceptible *Anopheles jeyporiensis* [2]. The cone bio-assays were performed at controlled temperature (27 ± 2 °C) and RH (75 ± 10%). Each time, a total of 600 field collected semi-gravid mosquitoes (5 mosquitoes per cone × 4 replicates × 5 positions per net) were exposed to LLIN cut pieces for 3 min and after 24 h, the mortality was recorded. Mosquitoes were exposed to untreated nets simultaneously and were used as negative controls.

### Data analysis

Analysis was performed using EPI DAT 3.1 software and Microsoft Excel 2013 (MS Office 2013, USA). The differences in the usage rate of LLINs by the respondents during the previous night among the three villages were compared using Chi square test. The washing of nets (at least once) among the users in three villages and the condition of the LLINs based on the pHI was also compared using Chi square test. A p-value of < 0.05 was taken as statistically significant.

## Results

### Usage rate of LLINs

A total of 130 persons residing in 130 households in 3 villages (each from TH, FH and PL) were surveyed. Altogether, 246 LLINs were distributed to the 130 households (Table 2). One LLIN was distributed for an average of 2.3 persons, during the net distribution campaign. Data on the usage of the existing LLINs by the household heads illustrated that among the 130 LLINs, 51% (n = 66) nets were used by the villagers during previous night and 49% (n = 64) nets were not in use (Table 2). The LLINs used previous night was found to be higher in PL village (61%) compared to FH (51%) and TH villages (30%) (Table 2). Significant difference was observed among the usage rate of LLINs in three villages (P = 0.028). Among the non-users of LLINs, 59% responded that the nets were ‘badly torn’ and 30% of the nets were not being used due to ignorance for not being aware of benefits. Sixty-four (49%) respondents told that they slept under LLINs during the previous 7 days whereas 53 (41%) people admitted that LLIN was not used during last 7 days. While, 70 to 76% respondents in three villages told that they are using the LLIN throughout the year, 24 to 26% of the users told that they use the LLINs seasonally, especially during rainy months. All the household heads told that they have used the LLIN at least once in a year. Nearly 90% users tuck the net under the mat while sleeping. Majority users (96%) users answered that they have never used LLIN away from house, whereas remaining 4% used their LLIN even when away from house (Table 3).

**Table 2:** General information on distribution of long-lasting insecticidal nets in study villages of Koraput district of Odisha, Eastern India

General information	Terrain			Total
Terrain	Hilltop	Foothill	Plain	
Total population of surveyed villages	456	882	1123	2461
Total no. of households (HHs)	112	201	220	533
No. of HHs selected	27	49	54	130
Year of distribution of LLIN	2017	2017	2017	
Total population in HHs surveyed	133	187	246	538
No. of LLINs provided to surveyed HHs	48	85	113	246
No. of LLINs inspected	27	49	54	130

LLINs long-lasting insecticidal nets, HHs households

**Table 3:** Usage and washing practices of long-lasting insecticidal nets in study villages

Net usage	TH (n = 27)	FH (n = 49)	PL (n = 54)	Total (n = 130)
No. of LLINs (%) available for sleeping	13 (48)	30 (61)	45 (83)	88 (68)
Throughout the year (%)	19 (70)	37 (76)	40 (74)	96 (74)
No of LLINs used previous night (%)	8 (30%)	25 (51%)	33 (61%)	66 (51%)
Seasonally (rainy) (%)	8 (30)	12 (24)	14 (26)	34 (26)
Tying inside house (%)	23 (86)	48 (98)	54 (100)	125 (96)
Washed the net	24 (89)	41 (84)	45 (83)	110 (85)
No. of nets dried under shade	24 (100)	40 (98)	41 (91)	105 (95)

TH, hilltop; FH, foothill; PL, plain; LLINs, long-lasting insecticidal nets

**Fig. 1:**
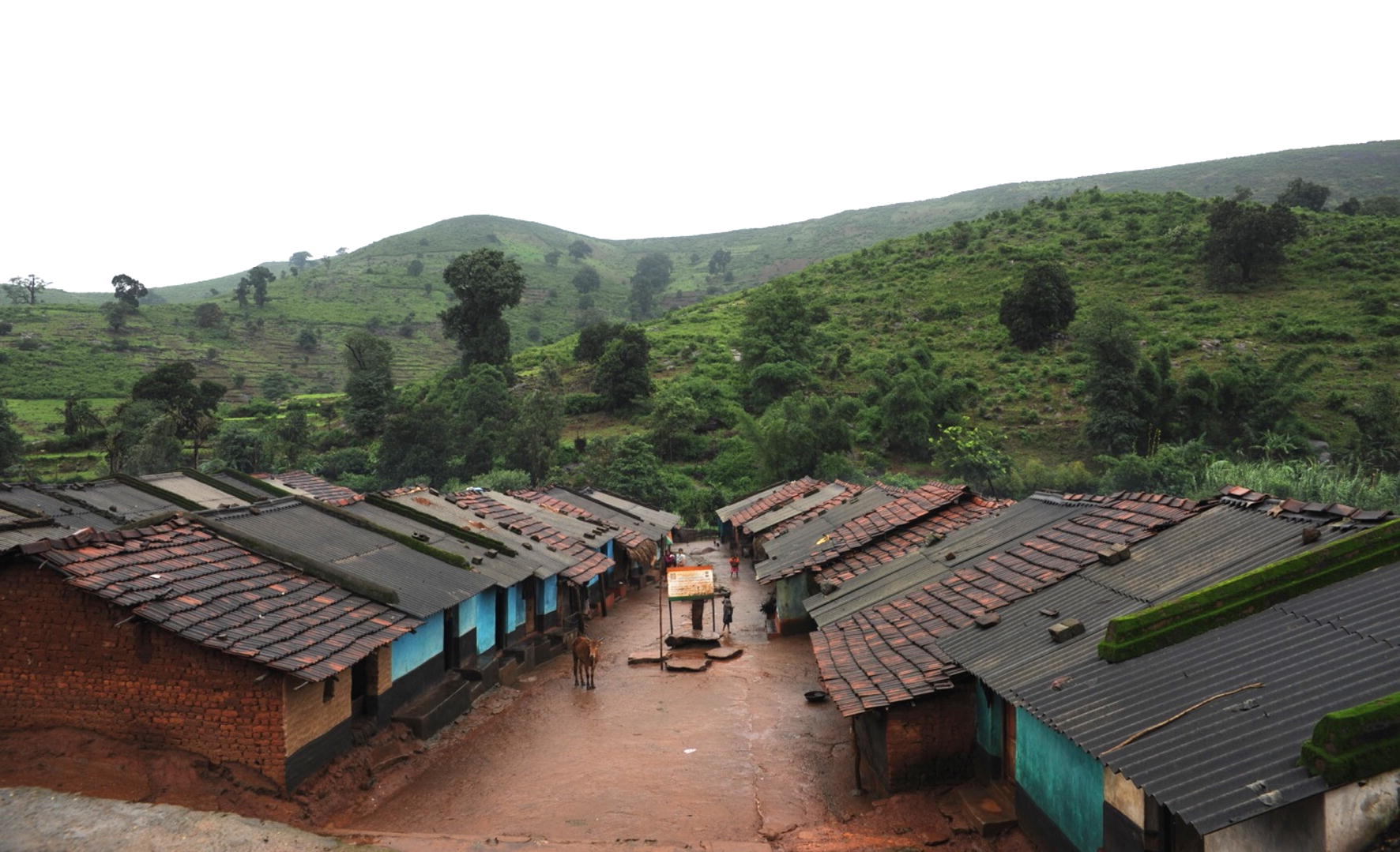
Picture showing terrain and housing pattern of foothill study village

During the surveys, 53 (40.8%) nets were visible from outside, while 32 (24.7%) nets were hanging loose over sleeping places, 18 (13.9%) nets were stored away, 16 (12.3%) nets were found folded and hung on rope, 9 (6.9%) nets found hanging tied as a knot and 1 (1%) net was used as a curtain and 1(1%) net had been lost and was not made available on the day of survey. No adverse effect was reported after the distribution of LLINs during their use. All the respondents informed the survey team that they had benefited from the use of supplied nets and they did not experience any mosquito bite during night time, unlike before the distribution of LLINs (Fig. 1).

### Washing practice of LLINs

Out of 130 observed nets, 110 (85%) nets were reported washed at least once within 30 months and 89%, 84% and 83% of LLINs were found washed in TH, FH and PL villages, respectively. No significant difference was observed in washing of nets (at least once) among the three villages (P-value = 0.860). All nets (100%) were washed with cold water, out of which 86 (78%) were washed by locally available detergent powder, while 1 (1%) net was washed with locally available soap and 23 (21%) nets were washed only with normal water without any soap. In all, 75% of the people did not soak the net before washing while 25% soaked the net for less than one hour. Of all users, 98% did not scrub the net hard, only 2% scrub and then beat the net with either a stick or hit it on a rock. Majority (95%) of nets were dried under the shade, while 5% nets were dried under sunlight (Table 3).

### Physical integrity of LLINs

Out of 130 holdings, one LLIN from foothill (FH) village holding was lost in the river on the day of survey. Hence, physical integrity was observed only of the 129 nets. A total of 77 nets (59.7%) were found to be with holes. In TH village, 78% nets were found with holes followed by 65% of nets in FH village and 46% of nets in PL village (Table 4). The total number of holes present in the nets with size 1, 2, 3 and 4 in the three villages are given in Table 4. The mean hole indices of LLINs was 1854.74, 1374.62 and 604.57 in TH, FH and PL villages, respectively (Table 5). Based on the pHI score, 74 (57%) nets were found ‘good’ (pHI is ≤ 64), 10 (8%) nets were in ‘serviceable’ condition (pHI is ≤ 642) and 45 (35%) nets were too torn and hence classified as having replaceable condition (pHI is > 642) (Table 6). Among the villages of three terrains, PL village had relatively higher proportion of nets in good condition (67%) in comparison to that of FH (54%) and TH (44%) villages; the difference was statistically significant among the three villages (P = 0.018).

**Table 4:** Distribution of holes in long-lasting insecticidal nets inspected in study villages

Village	No. of LLINs surveyed	No. of LLINs with holes (%)	Distribution of holes on net panels (%)			Mean no of open seams	Nets with any repairs (%)	Holes with any repairs (%)
			Roof	Upper	Lower			
TH	27	21 (79)	18	27	54	0.14	0.00	0.00
FH	48	31 (65)	8	22	70	0.00	4.08	1.11
PL	54	25 (46)	10	39	51	0.01	7.41	6.55

LLINs long-lasting insecticidal nets, TH, hilltop; FH, foothill; PL, plain

**Table 5:** Physical integrity of long-lasting insecticidal nets inspected in study villages

Hole size category	Hole size (cm)	Weight age of hole sizes	Hilltop (n = 27)		Foothill (n = 48)		Plain (n = 54)	
			No. of holes	Hole index	No. of holes	Hole index	No. of holes	Hole index
1	0.5 to 2	1	99	99	130	130	103	103
2	2 to 10	23	61	1403	82	1886	54	1242
3	10 to 25	196	65	12740	58	11368	27	5292
4	> 25	578	62	35836	91	52598	45	26010
Mean hole index			–	1854.7	–	1374.6	–	604.6

**Table 6:** Physical condition of distributed long-lasting insecticidal nets in study villages

Condition of nets (pHI)	TH (%) (n = 27)	FH (%) (n = 48)	PL (%) (n = 54)	Total (%) (n = 129)	Using of nets (%)	Nets not using (%)
Good (0–64 pHI)	12 (44)	26 (54)	36 (67)	74 (57)	57 (77)	17 (23)
Serviceable (65 to 642 pHI)	2 (7)	1 (2)	7 (13)	10 (8)	9 (90)	1 (10)
Too torn (> 643 pHI)	13 (48)	21 (44)	11 (20)	45 (35)	11 (24)	34 (76)
Total	27 (100)	48 (100)	54 (100)	129 (100)	77 (60)	52 (40)

LLINs, long-lasting insecticidal nets; pHI, proportionate hole index; TH, hilltop; FH, foothill; PL, plain

### Net survivorship and Attrition

A total of 246 LLINs were supplied to 130 selected holdings. After 30 months of distribution, 74.8% (n = 184) nets were physically present, whereas 25.2% (n = 62) nets were lost. The reasons for net losses were: disposal of nets due to wear and tear (19.1%), sold/stolen or given away (2.85%) and used for other purpose (3.25%). The rate of attrition was found to be higher in PL village (37.2) compared to FH (20.0) and TH (6.25) villages (Table 7). A total of 45 (93.75%), 68 (80%) and 71 (63.8%) LLINs were physically present in TH, FH and PL villages, respectively, during the survey.

**Table 7:** Attrition and net-survivorship at 30 months post-distribution of long-lasting insecticidal nets in study villages

Attrition	TH (%) (n = 48)	FH (%) (n = 85)	PL (%) (n = 113)	Total (%) (n = 246)
Attrition-1				
Wear and tear (disposed)	2 (4.16)	12 (14.11)	33 (29.20)	47 (19.10)
Attrition-2				
Sold/stolen/Gifted	0 (0.00)	2 (2.35)	5 (4.43)	7 (2.85)
Attrition-3				
Used for other purpose	1 (2.09)	3 (3.53)	4 (3.54)	8 (3.25)
Total attrition	3 (6.25%)	17 (20.0%)	42 (37.2%)	62 (25.2%)
LLINs present (survivorship)	45 (93.75%)	68 (80.0%)	71 (63.8%)	184 (74.8%)

LLINs, long-lasting insecticidal nets; TH, hilltop; FH, foothill; PL, plain

### Cone bio-assays

It was observed that, 50%, 33.3% and 16.6% of LLINs selected randomly for the bio-assays after 1 year of distribution were washed 5, 8 and 10 times, respectively. After second year, post distribution of LLINs, among the 6 selected nets, 33.3% (2 nos.) each were washed 15, 16 and 18 times, respectively. Similarly, at the end of 30 months, 33.3% (2 nos.) each were washed 13, 16 and 20 times, respectively. Bio-assays conducted after 1, 6, 18, 24 and 30 months of distribution of LLINs showed 100% mortality when exposed to *An. jeyporiensis*, indicating full retention of insecticidal efficacy in the LLINs even after 30 months of field use.

## Discussion

Malaria control programmes implementing LLINs as primary vector control strategy are required to monitor the survival of nets, fabric integrity and insecticidal efficacy so as to ensure timely replacement of nets [21]. Earlier, various studies have evaluated the effectiveness of different brands of LLINs in diverse socio-cultural and ecological areas in a research mode [3, 11, 22]. However, information is ought to be generated on the performance of the field used LLINs, their coverage, efficacy and usage pattern after the mass distribution by the national programme in Indian public health settings [19, 23, 24].

The current retrospective study has evaluated the performance of field used LLINs at operational level, 30 months after their distribution. The distribution pattern, usage, washing practices, durability, survivorship and attrition rate of LLINs used by the tribal population in 3 villages (one each in the TH, FH and PL) was assessed to corroborate the three-year durability of LLINs. As per the National Strategic Plan guidelines, distribution of LLINs should be in a proportion of 1.8 persons per net [17]. This study observed that during the distribution time, one net was given to an average of 2.3 persons and after 30 months of distribution, 25% of the nets were not available in the surveyed holdings. Therefore, nearly 42% population were out of the LLIN coverage at 30th month of their distribution. This was a major gap observed in the current study. Epidemiological success of LLIN tool can be achieved only by 100% coverage and at least 80% net usage rate by the community [25]. Therefore, authorities during the time of malaria elimination in India should take necessary steps to re-distribute LLINs to those who need them due to one of the various reasons identified during this study and to withdraw the nets that are no longer effective, in order to ascertain community protection against malaria and desired impact on its transmission.

The results of the current study showed that nearly 42% people were out of the LLIN coverage at 30 months after distribution and 51% of people slept under the LLIN last night. While combining the net usage rate and attrition rate, it could be concluded that around 30% of the population of the village only slept under a bed net last night, which could not provide a mass protection effect to the community. However, after distribution of LLINs, the majority of the villagers opined that the mosquito bite has reduced to a great extent. In this scenario, net replenishment with universal coverage is required to ensure full community protection.

It was observed in the current study that more than half (60%) of the nets had holes within 30 months of field use and nearly one-third of the nets (35%) were not in a usable condition. This clearly indicates that either the fabric quality of the LLIN was poor and did not satisfy the WHO criteria i.e., life of the net up to 36 months or mishandling of LLINs by the community.

Understanding LLINs attrition rate gives an important insight into survival, usage practice and quality of LLINs in terms of fabric integrity. In the current study, the major (75.8%) cause of attrition reported was the damage of nets due to wear and tear (true attrition); emphasizing that the distributed LLINs does not have a desired durability which could withstand the field conditions up to the prescribed 3 years. This is a serious concern which needs to be resolved by the LLIN manufacturing companies in future. However, it is a fact that, attrition rate in the current study was measured only at 30 months which prevented to know the mean survival time of the nets, which is important to know to indicate the right replacement rate. This was a limitation of the study. Many research studies have shown that most of the LLINs were either torn or were no longer present in the households before 3 years due to decreased durability, thereby suggesting that the serviceable life of LLIN was actually closer to two, rather than 3 years [6, 9, 26]. This study is one of a few studies that examined the durability of LLINs under operational conditions in India. Earlier studies were conducted in a research mode, therefore awareness on LLIN usage and washing practices and frequent visits of the research personnel might have influenced the results unlike observed during this study. The current study findings were a true reflection of the natural behaviour of the community on LLIN use without any prior information, education and communication (IEC) activities. A change in the distribution strategy by replacing mass distribution campaigns only once to that of a continuous distribution is necessary to sustain the huge gain achieved by the malaria elimination programme in the study district and elsewhere.

Based on the results of the current study, the state malaria control programme replenished the LLINs during 31st month of the distribution throughout the district which shows that if a follow up exercise is undertaken at 6 monthly intervals, simultaneous replacement can be done. Though WHO recommends conducting the bio-assays up to 36 months, but due to the loss in fabric integrity of LLINs and mass replenishment of LLINs, the test could not be conducted at 36 months.

The cone bio-assay results carried out showed that, all the nets were effective, in terms of insecticidal efficacy after 30 months of field use. This may be due to the reason that the washing frequencies of the nets were ≤ 20 washes, which might have retained the active ingredient and therefore net bio-efficacy. However, the loss of physical integrity of LLINs sooner than the expected period raises serious concern as 76% of the highly torn nets (> 643 pHI) were not used by the villagers.

## Conclusions

The elimination of malaria from Odisha state was planned during 2017 and targeted to achieve it in 2030 [17]. After, the mass distribution of LLINs, there was a marked decline in malaria incidences in the district as well as in the state and the malaria control programme exceeded the national target of 80% reduction in malaria morbidity and mortality within one and half years by the end of 2018 [18]. The achievements were impressive, being well above the National Framework for Malaria Elimination (NFME) target [27]. Yet, there are multiple challenges which may keep on increasing, as heading towards complete elimination. In the current study, attempt has been made to emphasize on some issues pertaining to universal coverage, usage rate and durability of LLINs that could be critical in successful malaria elimination in the state. This study is the first of its kind to report on the performance of LLINs under operational conditions in Odisha state by checking the physical integrity and the insecticidal potency of nets post deployment. The study revealed that universal coverage of LLINs was not achieved at the time of distribution. Attrition rate measured at 30 months of distribution was 25.0% and among the available nets, 35% were too damaged. This has collectively, however, become an area of major concern resulting in low net usage rate (51%). Therefore, the findings of the study are significant for NVBDCP to replenish the quality LLINs to sustain the gain achieved so far in reducing malaria morbidity and mortality.

## Data Availability

The datasets generated and/or analysed during the current study are available from the corresponding author on reasonable request.

## Abbreviations

LLIN: Long-lasting insecticidal net
NVBDCP: National Vector Borne Disease Control Programme
TH: Hilltop
FH: Foothill
PL: Plain
ITN: Insecticide-treated nets
CHC: Community Health Centre
ICMR-VCRC: Indian Council of Medical Research-Vector Control Research Centre
pHI: Proportionate hole index
NFME: National Framework for Malaria Elimination
